# Ingestion of whey protein and β-conglycinin exerts opposite effects on intestinal FGF15 and serotonin secretion in mice

**DOI:** 10.3389/fendo.2023.1080790

**Published:** 2023-01-27

**Authors:** Katsunori Nonogaki, Takao Kaji

**Affiliations:** Laboratory of Diabetes and Nutrition, Tohoku University, New Industry Creation Hatcher Center, Sendai, Miyagi, Japan

**Keywords:** whey protein, β-conglycinin, Fgf15, serotonin, bile acids

## Abstract

Farnesoid X receptor (FXR) and Takeda G protein-coupled Receptor 5 (TGR5), the intestinal bile acid (BA) receptors, regulate the gut-derived hormones including fibroblast growth factor 15/19 (FGF15/19) and serotonin (5-hydrooxytryptamine, 5-HT). Here we show that ingestion of whey protein isolate, a milk protein, significantly decreased expression of heteromeric organic solute transporter Ostα and Ostβ, which is the basolateral BA transporter in the enterocyte, and increased the expression of FXR and FGF15 in C57BL6J mouse ileum and plasma FGF15 levels. In addition, the ingestion of whey protein isolate significantly suppressed expression of hepatic cholesterol-7α hydroxylase (CYP7A1), which induces the primary BA synthesis, bile salt export pump (BSEP) and sodium-taurocholate cotransporting polypeptide (NTCP), which are the key transporters for the BA excretion and uptake in the liver, and genes involved in gluconeogenesis, and decreased the primary BAs including cholic acid, taurocholic acid, glycocholic acid, and taurochenodeoxycholic acid in the liver compared with controls. Moreover, ingestion of whey protein isolate significantly decreased the expression of TGR5, glucagon-like peptide 1 (GLP-1), and tryptophan hydroxylase1 (Tph1) in the small intestine, leading to decreases in plasma 5-HT and insulin levels. On the other hand, ingestion of the soy protein β-conglycinin significantly increased the expression of Ostα and Ostβ, and decreased the expression of FGF15 in the ileum and plasma FGF15 levels, leading to the increases in expression of hepatic CYP7A1, BSEP, NTCP, and genes involved in gluconeogenesis, and the primary BAs in the liver. Moreover, ingestion of β-conglycinin significantly increased the expression of intestinal TGR5, GLP-1, and Tph1, leading to increases in plasma 5-HT and insulin levels. These findings suggest that whey protein and β-conglycinin have opposite effects on intestinal FGF15 and 5-HT secretion in mice.

## Introduction

Bile acids (BAs) are nutrient sensors implicated to act as metabolic regulators that activate BA receptors, such as Farnesoid X receptor (FXR) and Takeda G protein-coupled Receptor 5 (TGR5), in the intestine (1–3). Activation of intestinal FXR, a BA-activated nuclear receptor, produces fibroblast growth factor 19 (FGF19) and its mouse ortholog FGF15, mainly in the enterocytes of the ileum (1–3). FGF15/19 is subsequently secreted into the portal vein and circulated to the liver, where it attenuates hepatic BA synthesis by suppressing the key enzyme cholesterol-7α hydroxylase (CYP7A1) and promotes gallbladder filling (1–4).

FGF15/19 is also suggested to act as a metabolic regulator (5). FGF15/19 is released postprandially from the small intestine and inhibits hepatic gluconeogenesis by inhibiting peroxisome proliferator-activated receptor γ coactivator protein-1α (PGC-1α) (6). Transgenic mice expressing FGF19 in muscle that are fed a high fat diet exhibit an increased metabolic rate, decreased serum insulin levels and adiposity, and improved glucose tolerance (7, 8). Thus, the FXR-FGF15/19 signaling pathway contributes to the regulation of hepatic BA synthesis in the enterohepatic cycle and energy metabolism. Intestinal BA transporters such as apical sodium-dependent bile acid transporter (ASBT) and heteromeric organic solute transporter Ostα-Ostβ, which are involved in the BA uptake and excretion in the enterocyte, regulate FGF15 expression in the mouse ileum (9).

In addition, TGR5 is a BA binding receptor expressed in the enteroendocrine cells (EE cells) of the intestine that stimulates the release glucagon-like peptide 1 (GLP-1), which induces postprandial insulin secretion by the pancreatic β-cells and stimulates serotonin (5-hydrooxytryptamine, 5-HT) release in the enterochromaffin cells (ECs) of small intestine (10).

Feeding on a high-fat diet increases hepatic FGF21 expression and plasma FGF21 and 5-HT levels in mice (11, 12), whereas it likely decreases intestinal FGF15/19 expression (13). Whey protein isolate is a milk protein obtained after the precipitation of casein during cheese production. We recently reported that ingesting whey protein isolate suppresses increases in hepatic FGF21 expression and plasma FGF21 levels, which precede hyperinsulinemia, insulin resistance, and hyperglycemia in mice fed a high-fat diet (12). On the other hand, the soy protein β-conglycinin reportedly has anti-obesity and glucose-lowering effects *via* increasing FGF21 in mice fed a high-fat diet (14). Ingesting β-conglycinin reportedly increases postprandial FGF21 levels in the plasma and hepatic FGF21 expression in mice fed a high-fat diet (14). Ingesting whey protein isolate and β-conglycinin may therefore have different effects on hepatic FGF21 expression and circulating FGF21 levels. Effects of ingesting whey protein isolate or β-conglycinin on intestinal FGF15 and 5-HT secretion, however, remain unclear.

In the present study, to investigate the effects of whey protein isolate or β-conglycinin on intestinal FXR-mediated FGF15 production, the FGF15 actions on the liver, and intestinal TGR5-mediated 5-HT production, we examined the effects of whey protein or β-conglycinin intake on expression of genes involved in the BA uptake and excretion, FXR and FGF15 in the ileum; plasma FGF15 levels; expression of hepatic genes involved in BA synthesis, the BA uptake and excretion, and gluconeogenesis; hepatic BAs; expression of intestinal TGR5, GLP-1, and tryptophan hydroxylase 1 (Tph1), which produces peripheral 5-HT; and plasma 5-HT and insulin levels in C57BL6J mice.

## Results and discussion

### Effects of whey protein isolate or β-conglycinin intake on the intestinal FXR and FGF15 expression and plasma FGF15 levels

**Figure 1:**
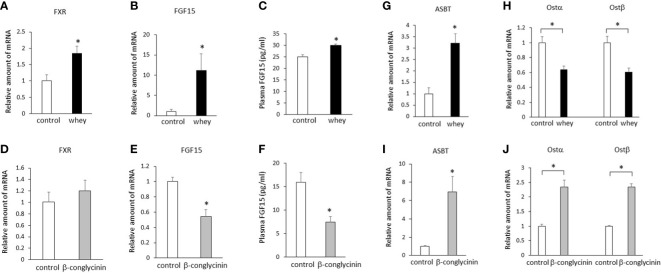
Effects of whey protein isolate (5 g/100 ml water) or β-conglycinin (5 g/100 ml water) intake on expression of FXR **(A, D)**, FGF15 **(B, E)**, ASBT **(G, I)**, Ostα, and Ostβ **(H, J)** in the ileum, and plasma FGF15 levels **(C, F)** in mice. The relative amount of mRNA is shown as fold-change of the mean value of the control group in C57BL6J mice fed a chow. Data are presented as the mean ± SEM (n = 6/group). *P < 0.05.

Ingestion of whey protein isolate for 3 days significantly increased expression of FXR and FGF15 in the ileum (**Figures 1A, B**) and plasma FGF15 levels (**Figure 1C**) compared with controls. On the other hand, ingestion of β-conglycinin for 3 days did not affect expression of FXR in the ileum (**Figure 1D**), but it significantly decreased expression of FGF15 (**Figure 1E**) in the ileum and plasma FGF15 levels (**Figure 1F**) compared with controls. These findings suggest that whey protein isolate intake upregulates intestinal FXR-mediated FGF15 production, whereas β-conglycinin intake downregulates intestinal FGF15 production without affecting FXR expression.

Either whey protein or β-conglycinin intake significantly increased expression of ASBT compared with controls in the ileum (**Figures 1G, I**). The ingestion of whey protein isolate significantly decreased expression of Ostα and Ostβ compared with controls in the ileum (**Figure 1H**), whereas ingestion of β-conglycinin significantly increased it (**Figure 1J**). These findings suggest that alterations of intestinal Ostα and Ostβ expression are related to the alterations of intestinal FGF15 expression induced by whey protein or β-conglycinin intake.

### Effects of whey protein isolate or β-conglycinin intake on hepatic BA synthesis and gluconeogenesis

**Figure 2:**
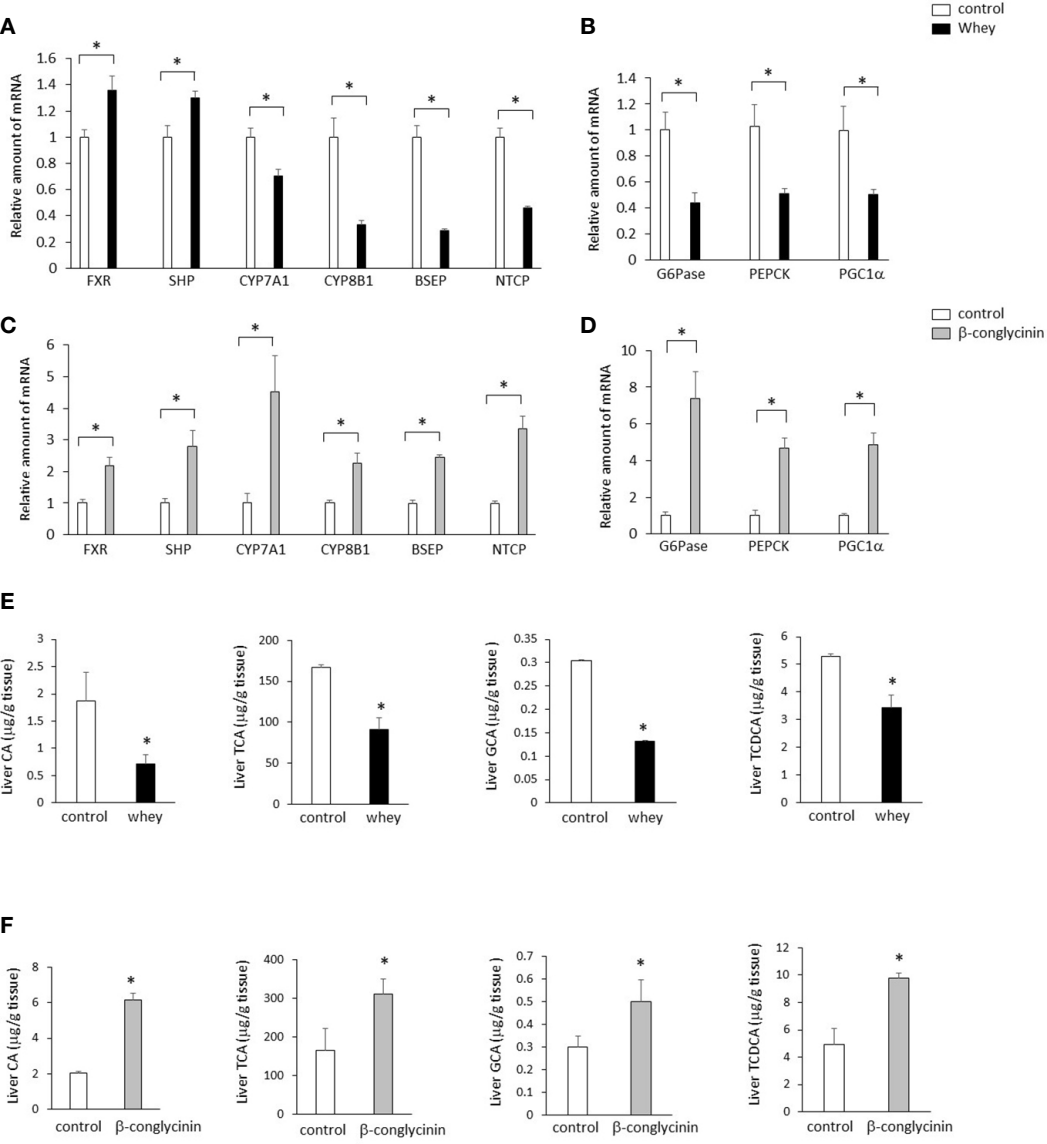
Effects of whey protein isolate (5 g/100 ml water) or β-conglycinin (5 g/100 ml water) intake on expression of hepatic FXR, SHP, CYP7A1, CYP8B1, BSEP and NTCP **(A, C)**, G6Pase, PEPCK and PGC-1α **(B, D)**, and hepatic CA, TCA, GCA and TCDCA **(E, F)** in mice. The relative amount of mRNA is shown as fold-change of the mean value of the control group in C57BL6J mice fed a chow. Data are presented as the mean ± SEM (n = 6/group). *P < 0.05.

The ingestion of whey protein isolate significantly increased expression of hepatic FXR and small heterodimer partner (SHP) and significantly decreased expression of hepatic CYP7A1, which increases BA synthesis (**Figure 2A**). In addition, the whey protein isolate intake significantly decreased bile salt export pump (BSEP) and sodium-taurocholate cotransporting polypeptide (NTCP), which are the key transporters for the BA excretion and uptake (**Figure 2A**). Moreover, the whey protein isolate intake significantly suppressed hepatic glucose 6-phosphatase (G6Pase), phosphoenolpyruvate carboxykinase (PEPCK) and PGC1α, which are involved in gluconeogenesis, compared with controls (**Figure 2B**). On the other hand, the ingestion of β-conglycinin for 3 days significantly increased the expression of hepatic FXR, SHP, CYP7A1, CYP8B1, BSEP and NTCP (**Figure 2C**), and G6Pase, PEPCK and PGC1α (**Figure 2D**) compared with controls.

Moreover, the ingestion of whey protein isolate significantly decreased cholic acid (CA), taurocholic acid (TCA), glycocholic acid (GCA) and taurochenodeoxycholic acid (TCDCA) in the liver compared with controls (**Figure 2E**), whereas the ingestion of β-conglycinin for 3 days significantly increased CA, TCA, GCA and TCDCA in the liver compared with controls them compared with controls (**Figure 2F**).

These findings suggest that whey protein isolate intake downregulates the CYP7A1-mediated BA synthesis and gluconeogenesis in the liver, whereas β-conglycinin intake upregulates them.

### Effects of whey protein isolate or β-conglycinin intake on intestinal TGR5, GLP-1, Tph1 expression and plasma 5-HT levels

**Figure 3:**
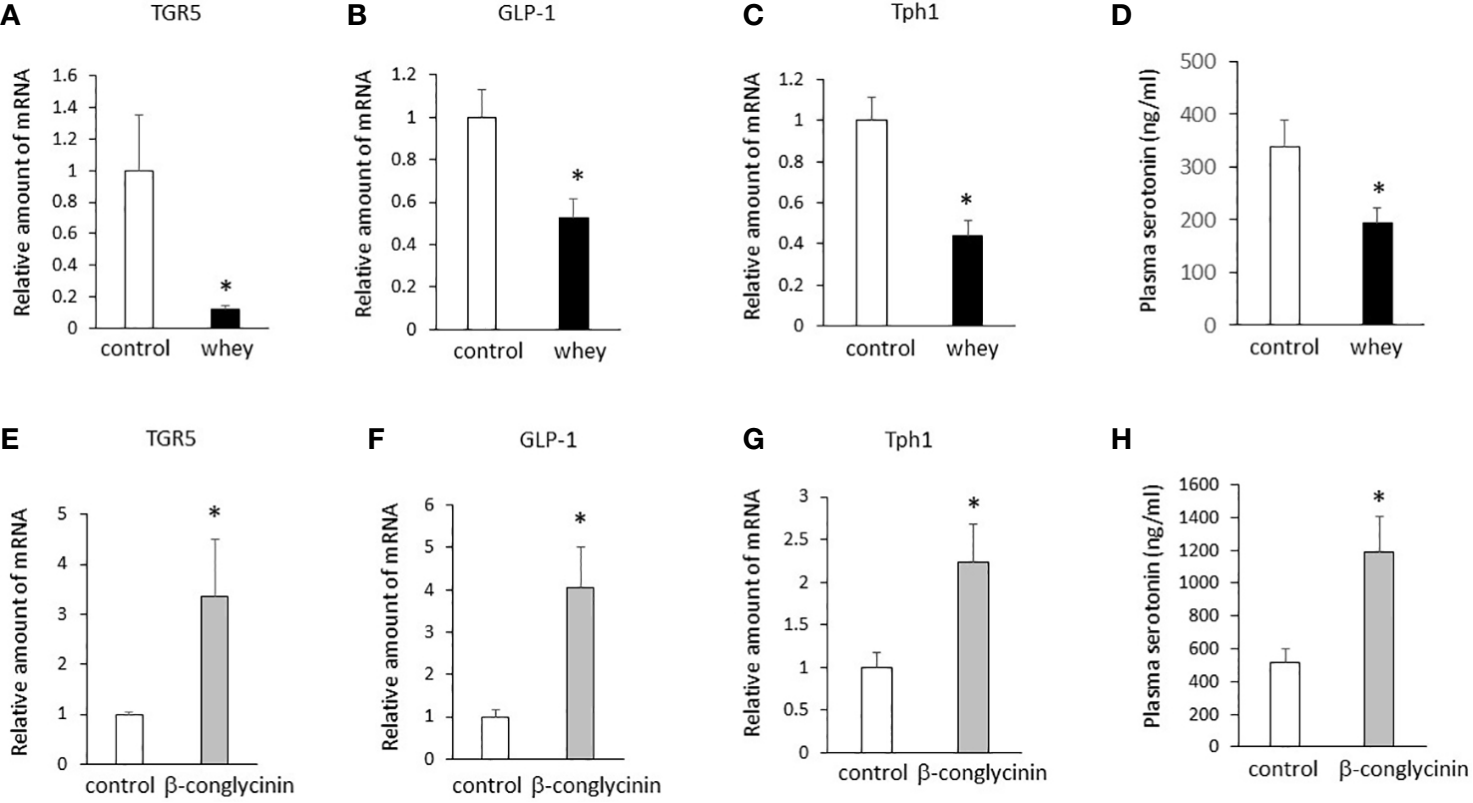
Effects of whey protein isolate (5 g/100 ml water) or β-conglycinin (5 g/100 ml water) intake on expression of TGR5 **(A, E)**, GLP-1 **(B, F)** and Tph1 **(C, G)** in the small intestine, and plasma 5-HT levels **(D, H)** in mice. The relative amount of mRNA is shown as fold-change of the mean value of the control group in C57BL6J mice fed a chow. Data are presented as the mean ± SEM (n = 6/group). *P < 0.05.

Moreover, ingestion of whey protein isolate for 3 days significantly decreased expression of TGR5, GLP-1 and Tph1 (**Figures 3A–C**) in the small intestine and decreased plasma 5-HT levels (**Figure 3D**) compared with controls. On the other hand, the ingestion of β-conglycinin for 3 days significantly increased expression of TGR5, GLP-1 and Tph1 (**Figures 3E–G**) in the small intestine and increased plasma 5-HT levels (**Figure 3H**) compared with controls. These findings suggest that whey protein isolate intake downregulates expression of TGR5, GLP-1 and Tph1 and plasma 5-HT levels in the small intestine, whereas β-conglycinin intake upregulates them.

### Effects of whey protein isolate or β-conglycinin intake on food intake, body weight, blood glucose and plasma insulin levels

**Figure 4:**
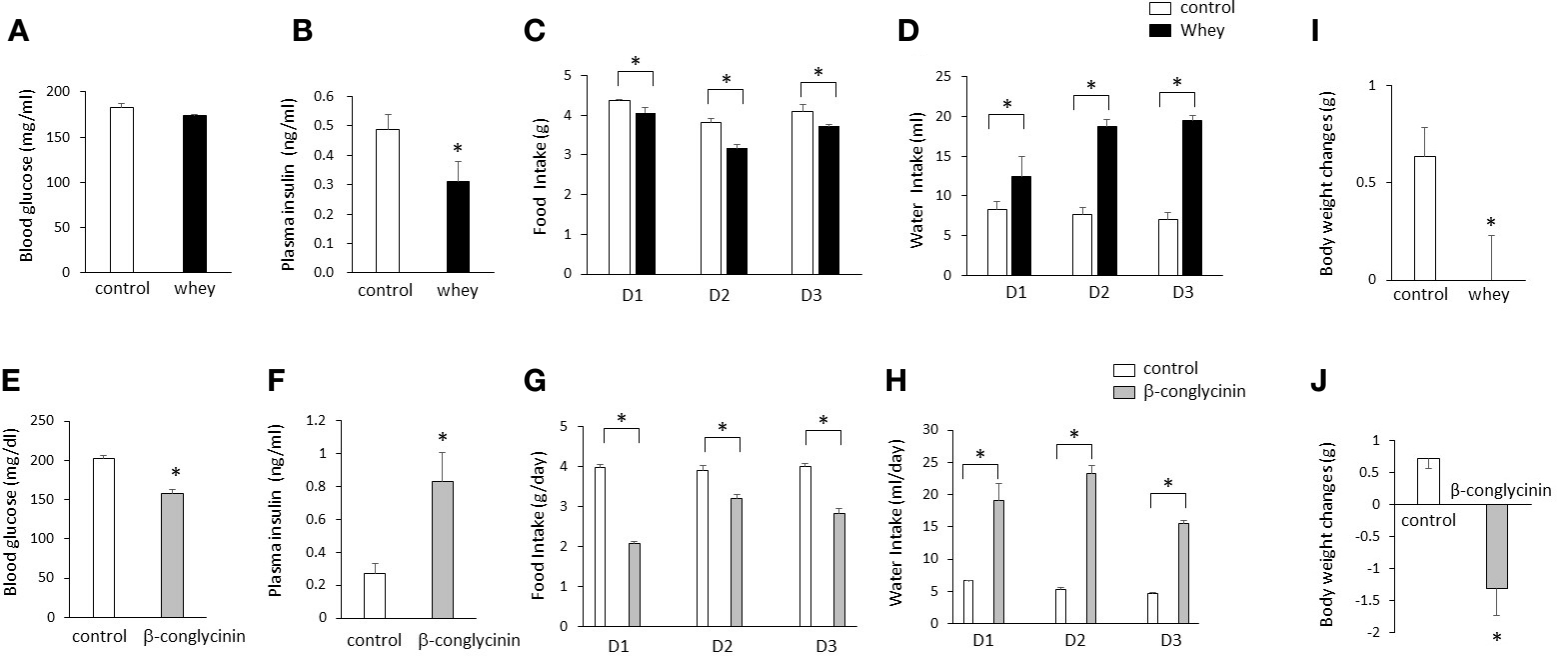
Effects of whey protein isolate (5 g/100 ml water) or β-conglycinin (5 g/100 ml water) intake on blood glucose levels **(A, E)**, plasma insulin levels **(B, F)**, daily food intake **(C, G)**, daily water intake **(D, H)** and body weight changes **(I, J)** in C57BL6J mice fed a chow. Basal body weights in mice were 19.9g ± 0.1g (controls) and 19.8 ± 0.1g (whey group), respectively. The relative amount of mRNA is shown as fold-change of the mean value of the control group in C57BL6J mice fed a chow. Basal body weights in mice were 19.0g ± 0.9g (controls) and 19.8 ± 0.7g (β-conglycinin group), respectively. Data are presented as the mean ± SEM (n = 6/group). *P < 0.05.

Although the ingestion of whey protein isolate had no significant effect on blood glucose levels (**Figure 4A**), it significantly decreased plasma insulin levels compared with controls (**Figure 4B**). The whey protein isolate intake significantly suppressed daily food intake for 3 days (**Figure 4C**) and body weight gain (**Figure 4I**) in mice, whereas it significantly increased daily water intake for 3 days (**Figure 4D**). The daily intake of whey protein was 1.0 ± 0 g on day 1, 1.28 ± 0.03 g on day 2, and 1.03 ± 0.02 g on day 3.

On the other hand, the ingestion of β-conglycinin significantly decreased blood glucose levels (**Figure 4E**) and increased plasma insulin levels compared with controls (**Figure 4F**). The β-conglycinin intake significantly suppressed daily food intake for 3 days (**Figure 4G**) and body weight gain (**Figure 4J**) in mice, whereas it significantly increased daily water intake for 3 days (**Figure 4H**). The daily intake of β-conglycinin was 0.95 ± 0.13 g on day 1, 1.16 ± 0.06 g on day 2, and 0.78 ± 0.02 g on day 3.

These findings suggest that the ingestion of whey protein isolate downregulates plasma insulin levels, whereas the ingestion of β-conglycinin upregulates them, and that the changes in plasma insulin levels induced by whey protein isolate and β-conglycinin are independent of food intake and body weight.

The apical and basolateral BA transporters in the enterocyte regulate FGF15 expression in the ileum and CYP7A1 expression and BA synthesis in the liver (9). Genetic ablation of Ostα increases expression of FGF15 in the ileal enterocyte and decreases CYP7A1 expression and BA synthesis in the liver, whereas genetic ablation of ASBT exerts the opposite effects on them (9). Our results of the present study demonstrated that the alterations of Ostα and Ostβ expression induced by whey protein isolate or β-conglycinin intake are related to the alterations of FGF15 expression in the ileum. Thus, the altered expression of Ostα and Ostβ in the ileum may have a critical role of the alterations of FGF15 expression and secretion induced by whey protein or β-conglycinin intake.

Feeding induces the expression of hepatic CYP7A1, which is the rate limiting enzyme in the synthesis of primary BAs, whereas fasting suppresses its expression (1). The present findings demonstrated that CYP7A1 expression and primary BA synthesis in the liver were downregulated by the ingestion of whey protein isolate, whereas they were upregulated by the ingestion of β-conglycinin. Because body weight and daily food intake were decreased in mice fed either whey protein isolate or β-conglycinin, the effects of whey protein and β-conglycinin on hepatic CYP7A1 expression and BA synthesis were independent of the changes in food intake and body weight.

Two pathways, the intestinal FXR-FGF19/15 signaling and hepatic FXR-SHP signaling pathways, reportedly contribute to the regulation of hepatic CYP7A1-mediated BA synthesis (1–3). Although ingestion of either whey protein isolate or β-conglycinin upregulated hepatic FXR and SHP expression, ingestion of whey protein isolate and β-conglycinin exerted opposite effects on hepatic CYP7A1 expression and BA synthesis in relation to alterations in the intestinal FGF15 expression, plasma FGF15 levels, and hepatic gluconeogenesis. These findings suggest that alterations of hepatic CYP7A1 expression and BA synthesis induced by a protein diet likely depend on FGF15 signaling rather than hepatic FXR-SHP signaling *in vivo*.

TGR5, BA binding receptor, is expressed in EE cells of the small intestine and promotes GLP-1 synthesis and release from EE cells of the small intestine (10). GLP-1 reportedly stimulates 5-HT release from the ECs of the small intestine (10). Thus, BA may upregulate 5-HT synthesis and release from the ECs through the TGR5-GLP-1 axis in EE cells of the small intestine. Our results demonstrated that the ingestion of whey protein isolate downregulated the expression of TGR5, GLP-1, and Tph1 in the small intestine and plasma 5-HT levels, whereas the ingestion of β-conglycinin upregulated them. Thus, the decreases in hepatic BA synthesis induced by ingestion of whey protein isolate may interfere with the TGR5-GLP-1 axis in EE cells, leading to the suppression of 5-HT synthesis in EC cells of the small intestine and circulating 5-HT levels. Moreover, our results demonstrated that changes in the plasma insulin levels are likely related to changes in the plasma 5-HT levels induced by whey protein isolate or β-conglycinin intake, whereas they are negatively related to changes in FGF15 levels, suggesting that the enterohepatic cycle of BAs, including FGF15, contribute to the regulation of protein intake-induced 5-HT and insulin secretion.

Plasma 5-HT levels are increased in obesity, type 2 diabetes and NAFLD in rodents and humans (11). Genetic, pharmacologic and nutrimental suppression of circulating 5-HT levels *via* Tph1 can protect against high-fat diet-induced metabolic diseases in mice (12, 15, 16). Circulating FGF21 levels are increased in obesity, type 2 diabetes and NAFLD (11, 12), whereas circulating FGF15/19 levels are decreased in them (17). Circulating FGF21 levels are positively correlated to insulin resistance in obesity, whereas circulating FGF19 levels are negatively correlated to it (17).

Bariatric surgery such as Roux-en-Y gastric bypass or vertical sleeve gastrectomy for obesity and type 2 diabetes likely decreases circulating FGF21 levels and increases circulating FGF15/19 levels (18–21). Our previous report demonstrated that the ingestion of whey protein isolate decreases plasma FGF21 and 5-HT levels in mice fed either chow diet or a high-fat diet (12). The decreases in plasma FGF21 and 5-HT levels induced by whey protein intake are associated with decreased insulin resistance and improved glucose tolerance in mice fed a high fat diet (12). Moreover, our present study demonstrated that the ingestion of whey protein isolate increased FGF15 expression in the ileum and plasma FGF15 levels. Taken together, the effects of whey protein intake on circulating FGF21 and FGF15/19 levels may be similar to the effects of bariatric surgery. Further studies will be needed in future.

In summary, these findings suggest that whey protein intake increases intestinal FGF15 secretion and decreases intestinal 5-HT secretion in mice, whereas β-conglycinin intake has the opposite effects on them.

## Materials and methods

### General procedures

Male C57BL6J mice (5 weeks old) were purchased from Japan CLEA. The mice were individually housed in cages with free access to water and chow pellets in a light- and temperature-controlled environment (12 h on/12 h off, lights on at 08:00; 20–22°C).

In the first experiment, 5-week-old C57BL6J mice were fed a chow diet with or without whey protein isolate (5 g/100ml water) for 3 days (days 10 through 13).

In the second experiment, 5-week-old C57BL6J mice were fed a chow diet with or without β-conglycinin (5 g/100ml water) for 3 days (days 10 through 13).

Daily water intake and food intake and body weight changes were determined. Finally, the animals were decapitated in fed state and blood was obtained for the measurement of blood glucose, plasma FGF15, 5-HT, and insulin levels. The liver, ileum and small intestine were dissected for determining mRNA levels.

The experiments were performed between 14:00-16:00. Whey protein isolate (Provon 190; protein 93%, water 3.5%, lipid 0.4%, and ash 2.8%; pH 6.0-6.5) was obtained from Glanbia Nutritionals (Niseikyoeki Co, Japan) as described previously (12). β-Conglycinin was kindly given by Fuji Oil Co, LTD, Japan.

The animal studies were conducted in accordance with the institutional guidelines for animal experiments at Tohoku University Graduate School of Medicine and all experimental protocols were approved by the institutional ethics committee at Tohoku University.

### Blood chemistry

Whole blood was mixed with EDTA-2Na (2 mg/ml) and aprotinin (500 kIU/ml) to determine the plasma levels of FGF15, 5-HT, and insulin. Plasma insulin were measured by enzyme-linked immunosorbent assay (mouse Insulin ELISA Kit [TMB], AKRIN-011T, Shibayagi, Gunma, Japan) as described previously (12). Plasma levels of FGF15 were measured by enzyme-linked immunosorbent assay (mouse FGF15 ELISA kit, CSB-EL522052MO, WUHAN HUAMEI BIOTECH Co). Plasma 5-HT levels were measured by enzyme-linked immunosorbent assay (mouse 5-HT; BA E-5900, Labor Diagnostika Nord, Nordhorn, Germany). Blood glucose levels were measured using glucose strips (blood glucose monitoring system; Accu-Check, Roche Diagnostics, Tokyo, Japan).

### Real-time quantitative reverse transcription–polymerase chain reaction

Total RNA was isolated from mouse liver using the RNeasy Midi kit (Qiagen, Hilden, Germany) according to the manufacturer’s instructions. cDNA synthesis was performed using a Super Script III First-Strand Synthesis System for RT-PCR Kit (Invitrogen, Rockville, MD) with 1 μg total RNA. cDNA synthesized from total RNA was evaluated in a real-time PCR quantitative system (LightCycler Nano Instrument Roche Diagnostics, Mannheim, Germany). The primers were listed in **Table 1**.

**Table 1:** The primers of RT-PCR were listed.

GENES		SEQUENCE
**FGF15**	Sense	ACGGGCTGATTCGCTACTC
	Antisense	TGTAGCCTAAACAGTCCATTTCCT
**FXR**	Sense	CCCCTGCTTGATGTGCTAC
	Antisense	CGTGGTGATGGTTGAATGTC
**SHP**	Sense	CGATCCTCTTCAACCCAGATG
	Antisense	AGGGCTCCAAGACTTCACACA
**CYP7A1**	Sense	AGCAACTAAACAACCTGCCAGTACTA
	Antisense	GTCCGGATATTCAAGGATGCA
**GLP-1**	Sense	TGAGATGAGCACCATTCTGGA
	Antisense	TCCGCAGAGATGTTGTGAAGA
**TGR5**	Sense	TCCTGTCAGTCTTGGCCTATGA
	Antisense	GGTGCTGCCCAATGAGATG
**Tph1**	Sense	ACCATGATTGAAGACAACAAGGAG
	Antisense	TCAACTGTTCTCGGCTGATG
**G6Pase**	Sense	TGCAAGGGAGAACTCAGCAA
	Antisense	GGACCAAGGAAGCCACAATG
**PEPCK**	Sense	AGCGGATATGGTGGGAAC
	Antisense	GGTCTCCACTCCTTGTTC
**PGC1α**	Sense	GTAGCGACCAATCGGAAATC
	Antisense	CTAGCAAGTTTGCCTCATTCTC
**BSEP**	Sense	CTGCCAAGGATGCTAATGCA
	Antisense	CGATGGCTACCCTTTGCTTCT
**NTCP**	Sense	ATGACCACCTGCTCCAGCTT
	Antisense	GCCTTTGTAGGGCACCTTGT
**ASBT**	Sense	TGGGTTTCTTCCTGGCTAGACT
	Antisense	TGTTCTGCATTCCAGTTTCCAA
**Ostα**	Sense	TACAAGAACACCCTTTGCCC
	Antisense	CGAGGAATCCAGAGACCAAA
**Ostβ**	Sense	GTATTTTCGTGCAGAAGATGCG
	Antisense	TTTCTGTTTGCCAGGATGCTC
**CYP8B1**	Sense	CTAGGGCCTAAAGGTTCGAGT
	Antisense	GTAGCCGAATAAGCTCAGGAAG
**β-actin**	Sense	TTGTAACCAACTGGGACGATATGG
	Antisense	GATCTTGATCTTCATGGTGCTAGG

The relative amount of mRNA was calculated using β-actin mRNA as the invariant control. Data are shown as fold-change of the mean value of the control group as described previously (12).

### BA analysis

BA analysis was performed using a facility service at LSI Medience Corporation, contract clinical trial company. (Tokyo, Japan). Briefly, approximately 100 mg of liver tissue was transferred to disruptor tubes supplied by Yasui Kikai (Osaka, Japan) and shaken with iron cones cooled in liquid nitrogen. The tissue powders were suspended with 1 mL of water and 4 mL of methanol. After mixing using a shaker for 15 min, the samples were centrifuged at 1000 × g for 15 min at room temperature. The supernatants were analyzed by liquid chromatography-tandem mass spectrometry (Nexera X2 LC0AD, 8050, Shimadzu, Kyoto, Japan) equipped with reverse phase LC column (InfinityLab Poroshell 120 EC-C18 2.7 mm, 2.1 mm ×150 mm, Agilent Technologies, Santa Clara, CA). The data were analyzed by LabSolutions (Shimadzu, Kyoto, Japan). The peak areas were normalized by internal standards, and each bile acid concentration was obtained using a standard curve (22).

### Statistical methods

Data are presented as mean ± SEM (n= 6). Comparisons between two groups were performed using Student’s t-test. A P value of less than 0.05 was considered statistically significant.

## Data availability statement

The raw data supporting the conclusions of this article will be made available by the authors, without undue reservation.

## Ethics statement

The animal study was reviewed and approved by Tohoku University.

## Author contributions

KN designed the study, performed the experiments, interpreted all analyses, generated all figures and tables, and wrote the manuscript. TK performed the experiments and interpreted all analysis. All authors contributed to the article and approved the submitted version.
